# A Miniaturized Circularly-Polarized Antenna for In-Body Wireless Communications

**DOI:** 10.3390/mi10010070

**Published:** 2019-01-19

**Authors:** Yi Fan, Xiongying Liu, Jiming Li, Tianhai Chang

**Affiliations:** 1 School of Electronics and Information, Guangdong Polytechnic Normal University, Guangzhou 510665, China; hnanfy@163.com; 2 School of Electronic and Information Engineering, South China University of Technology, Guangzhou 510641, China; hnanlxy@163.com (J.L.); thchang@scut.edu.cn (T.C.)

**Keywords:** circular polarization, implantable antenna, reactive loading, slow wave effect

## Abstract

A novel miniaturized single-fed circularly-polarized (CP) microstrip patch antenna operating in the Industrial, Scientific, Medical (ISM) band of 2.40–2.48 GHz, is comprehensively proposed for implantable wireless communications. By employing reactive loading in the arrow-shaped slotted patch to form slow wave effect and embedding V-shaped slots into patch to lengthen the current path, the proposed implantable antenna is minimized with the overall dimensions of 9.2 mm × 9.2 mm × 1.27 mm. The radiation patterns of the proposed antenna illustrate the performance of left-handed circular polarization. The simulated results show that an impedance bandwidth of 7.2% (2.39–2.57 GHz) and an axial ratio bandwidth of 3.7% (2.39–2.48 GHz) at the ISM band are achieved, respectively. Ex vivo measured results are in good agreement with the corresponding simulated ones.

## 1. Introduction

Implantable medical devices (IMDs) have increasingly caught the attention of the scientific community due to their wireless capabilities of detecting bio-medical information and transmitting health data much more flexibly and conveniently than traditional wired sensors placed exterior to the body [1,2,3]. These devices have been widely adopted in many applications including neural recording [4], glucose monitoring [5], and intracranial pressure monitoring [6], etc. 

Implantable antennas act as a key factor to assure wireless communications between the implantable devices and the external equipment [7], and have been assigned at the industrial, scientific, and medical (ISM) bands with the operating frequency ranges of 433.1–434.8 MHz [8,9], 902–928 MHz [10], and 2.40–2.48 GHz [11,12]. Additionally, the 402–405 MHz medical implant communication services (MICS) band [13] and the 1395–1400 MHz wireless medical telemetry services (WMTS) band [14] are also designated for the implantable antennas. Implantable devices used for the biomedical telemetry are typical microsystem and therefore cannot accommodate large antennas. Due to the relatively short electromagnetic wavelength, the implantable antenna working at the ISM higher frequency band of 2.40–2.48 GHz is adopted by some researchers, making the dimension electrically small enough. Moreover, considerable design efforts, such as employing high-permittivity dielectric substrates [15], loading shorting pins to connect the patch and the ground [16], and extending the current flow path on the patch surface [17], have been made to realize the compact dimension of the implantable antennas. Many crucial factors such as biocompatibility, specific absorption rate (SAR), far-field radiation, and operating bandwidth, should be also considered during the designing of implantable antennas.

The planar inverted-F antenna (PIFA) was demonstrated as a useful prototype in the design of implantable antennas because of its structural simplicity and low profile [18]. The monopole antenna with the advantages of omnidirectional radiation pattern and wide bandwidth has been integrated with the implantable system [19]. In [20], a differentially-fed dual-band flexible antenna was proposed for ingestible capsule system. Nevertheless, the above-mentioned antennas are linearly polarized and dependent on the relative orientations between the transmitters and the external receivers.

A circularly-polarized (CP) antenna is preferred for the implantable devices because it can reduce multipath distortion and provide flexible mobility, compared with a linearly polarized antenna. However, only a few works focus on the miniaturized CP implantable antennas. Capacitive loadings were introduced in a square patch with a central squared slot to realize circular polarization for an implantable antenna [21]. An axial-mode multilayer helical circularly-polarized implantable antenna for ingestible capsule endoscope system was presented in [22]. Additionally, by cutting rectangular slots into the patch and adding open stubs in the annular ring, a CP bio-friendly implantable annular ring antenna was realized in [23].

A miniaturized CP squared patch antenna for implantable devices at the ISM band of 2.40–2.48 GHz was preliminarily introduced in [24]. After that, future works have been carried out. In this article, an in-depth study on the working principle and a more detailed analysis on the performance of the proposed antenna are presented while a proof-of-concept fabricated prototype was fully characterized, verifying the good performance of the proposed antenna topology. Through introducing reactive loading, slow wave effect is formed on the radiator, making the dimension of the proposed antenna compact. 

The contents of this article are organized as follows: In Section 2, a simulation environment is set up, the geometry of the proposed implantable antenna is described, and the simulation results are studied. In Section 3, the miniaturization of working mechanism, CP properties, and parameter analysis are given. Section 4 presents the measured results before a useful conclusion is made in Section 5.

## 2. Antenna Design and Simulation

### 2.1. Simulation Environment

**Figure 1:**
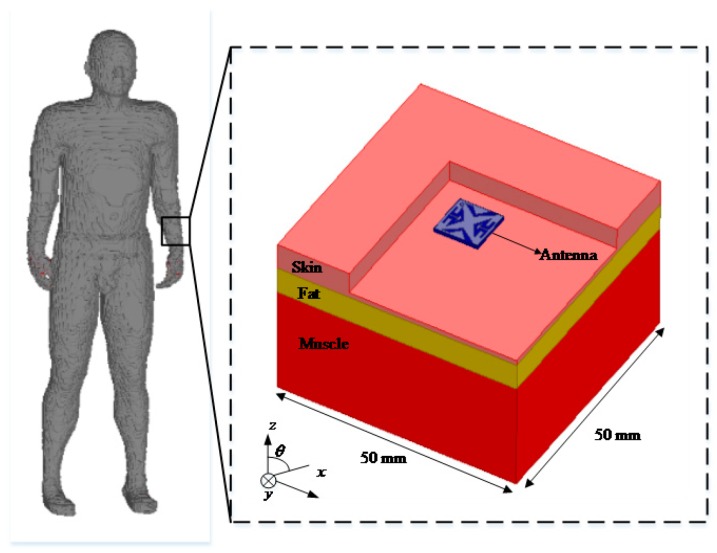
Simulation environment of the proposed antenna.

As shown in Figure 1, the proposed antenna is simulated in a three-layer tissue numerical model with the dimensions of 50 mm × 50 mm × 58 mm that imitates the real human environment. The human tissue is composed of skin, fat, and muscle. The electrical properties of the tissues vary with frequency. Table 1 lists the values of dielectric properties for skin, fat, and muscle in the simulated model at 2.45 GHz. To be closer to external devices and reduce the path loss in the tissue, the proposed antenna is implanted in a depth of 2 mm from the top of skin. The simulated tool employs ANSYS High Frequency Structure Simulator (HFSS) software (v.13, Ansys Inc., Canonsburg, PA, USA) for modeling, optimizing, and analyzing.

### 2.2. Geometry of the Proposed Circularly-Polarized Antenna

**Figure 2:**
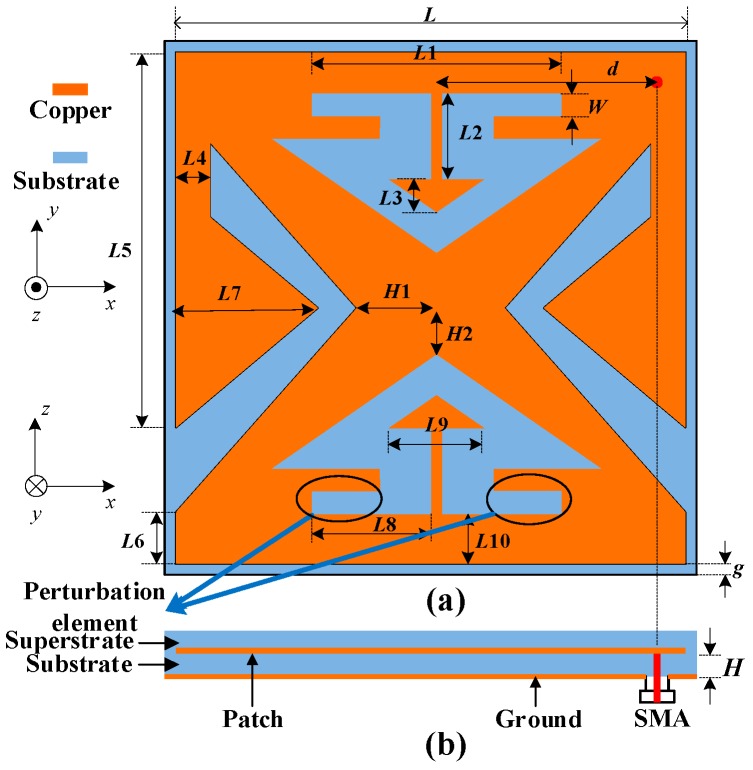
Geometry of the proposed antenna at: (**a**) Top view; and (**b**) side view.

The configuration of the proposed implantable antenna is demonstrated in Figure 2, the dimensions of the patch are fixed to 9.0 mm × 9.0 mm with a ground plane of 9.2 mm × 9.2 mm. To achieve the miniaturization, the antenna is manufactured on a Rogers 3010 substrate with a high dielectric constant of *ε_r_* = 10.2 and a low loss tangent of tan *δ* = 0.0035, covered by a layer of superstrate with the same material as the substrate, each with a thickness of *H* = 0.635 mm. The superstrate is utilized to separate human tissues from the conducting patch of the proposed antenna and to enhance the matching with the around inner tissues. In order to avoid shorting and to relieve mismatching, the proposed antenna should be wrapped by a thin film of biocompatible materials alumina (*ε_r_* = 9.2, tan *δ* = 0.008). The 50-Ω coaxial cable feeding point is welded at the position (*d*, *d*) along the center of diagonal of the patch. Two small triangle patches are embedded on the upper and lower sides of the proposed antenna and connected with the main patch through two high impedance lines. Additionally, V-shaped slots are embedded into the left and right side of the proposed antenna. It should be noted that perturbation slots are cut to optimize the axial ratio (AR). Table 2 lists the detailed values of the geometrical dimension after the optimization with the aid of ANSYS HFSS.

### 2.3. Simulated Results

**Figure 3:**
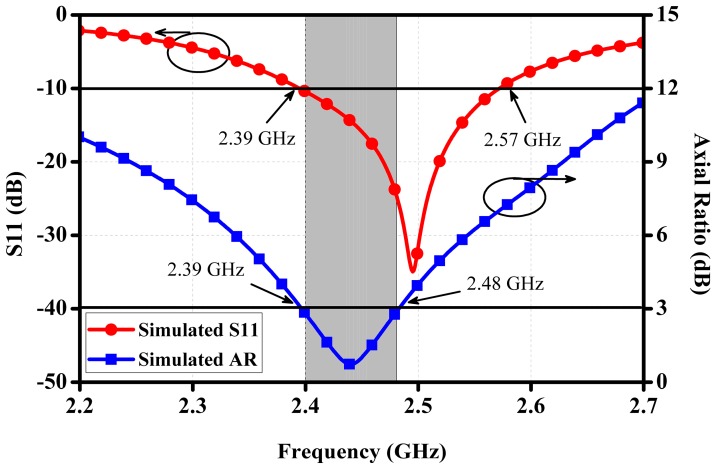
Simulated reflection coefficient and axial ratio (AR) varying with frequency.

Figure 3 illustrates the simulated reflection coefficient together with the axial ratio (AR) of the proposed antenna in main radiation direction towards the outside of human body. The simulated impedance bandwidth is covered from 2.39 GHz to 2.57 GHz with S11 less than −10 dB while the AR bandwidth can be extended from 2.39 GHz to 2.48 GHz with AR below 3 dB.

**Figure 4:**
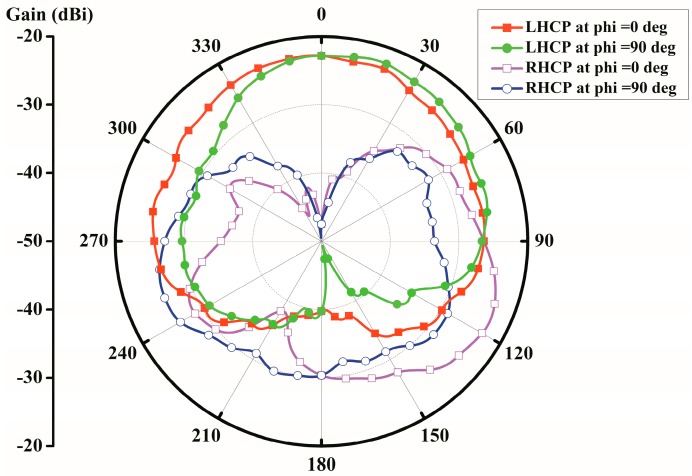
Simulated radiation patterns at 2.45 GHz.

Figure 4 depicts the simulated far-field gain radiation patterns of the proposed antenna in two principal planes (i.e., *xy*-plane and *xz*-plane) at 2.45 GHz. Its maximum left-handed circular polarization (LHCP) radiation is towards the antenna’s boresight at theta = 0°, that is the off-body direction as desired. Due to the fact that the proposed antenna is very compact and implanted in the lossy tissue with the limited space, different from the conventional antenna operating in free space, the peak realized gain is −24.8 dBi at 2.45 GHz.

## 3. Antenna Analysis

### 3.1. Miniaturization of the Proposed Antenna

**Figure 5:**
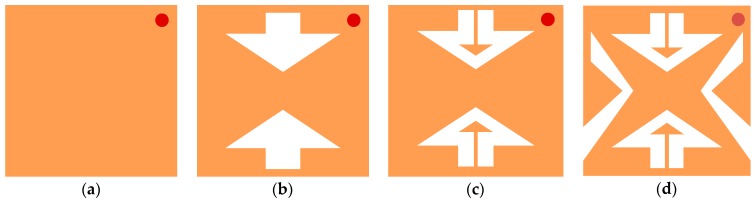
Evolving structures of the proposed antenna in: (**a**) Case 1; (**b**) Case 2; (**c**) Case 3; and (**d**) Case 4.

In order to investigate the mechanism of miniaturization, the corresponding topology of the antenna is evolved from Case 1 to Case 4 by subsequently cutting slots and loading patch into a conventional square patch antenna, as shown in Figure 5. Here, all antennas keep the fixed sizes of 9.2 mm × 9.2 mm and the simulating settings are similar.

**Figure 6:**
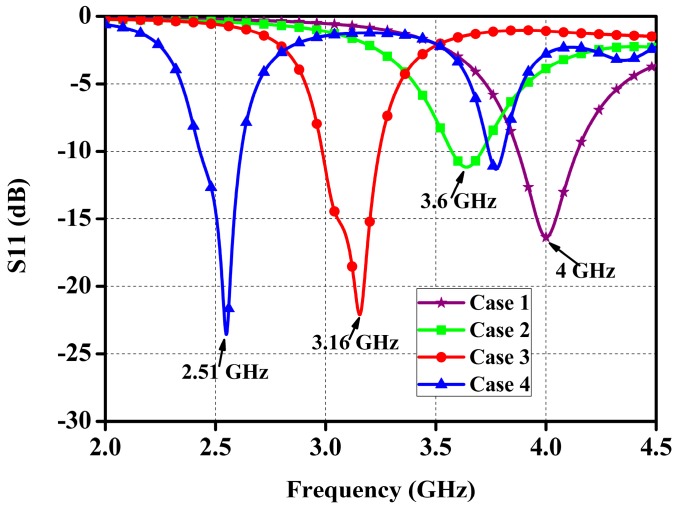
Simulated S11 of four cases embedded in the same phantom.

With reference to Figure 5, Case 1 is a conventional square patch antenna with the feeding port at the upper right diagonal, Case 2 is obtained by etching an arrow-shaped slot in the upper and lower parts of the patch. In Case 3, two small triangle patches are respectively loaded on the arrow-shaped slot of Case 2 and connected with the main patch through two high impedance lines. As shown in Figure 6, the resonant frequency of 4 GHz in Case 1 shifts down to 3.6 GHz in Case 2, then converts to 3.16 GHz in Case 3, indicating that a 21% of miniaturization can be achieved.

**Figure 7:**
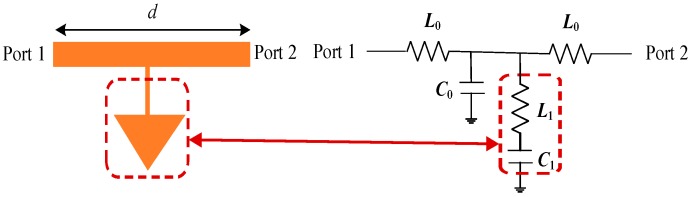
Transmission line model with LC loadings.

Theoretically, an antenna can be equivalent to a transmission line, as shown in Figure 7, which is characterized by a series inductance *L*_0_ and a shunt capacitance *C*_0_ per unit length. When the antenna is loaded with patch through a high impedance lines, the miniaturization of the proposed antenna can be achieved by taking advantage of the principle of slow waves [25]. The mechanism can be understood through calculating the propagation velocity as:

(1) $$\nu_{p}=\frac{1}{\sqrt{L_{0}C_{0}}}=\frac{c}{\sqrt{\varepsilon_{eff}}}=\lambda_{g}f$$

According to Equation (1), by adding triangle patches (equivalent to a capacitance *C*_1_) and high impedance line (equivalent to an inductance *L*_1_), total equivalent capacitance and/or inductance are increased, subsequently propagation velocity becomes slower, resulting in waveguide wavelength smaller when the frequency remains unchanged.

To further miniaturize the proposed antenna, V-shaped patches are etched into the left and right sides of Case 3, lengthening the current path, as established in Case 4 of Figure 5d. Compared with Case 1, the corresponding resonate frequency of Case 4 is shifted down from 4 GHz to 2.51 GHz, demonstrating that 37.3% of miniaturization is achieved.

### 3.2. CP property of the Antenna

**Figure 8:**
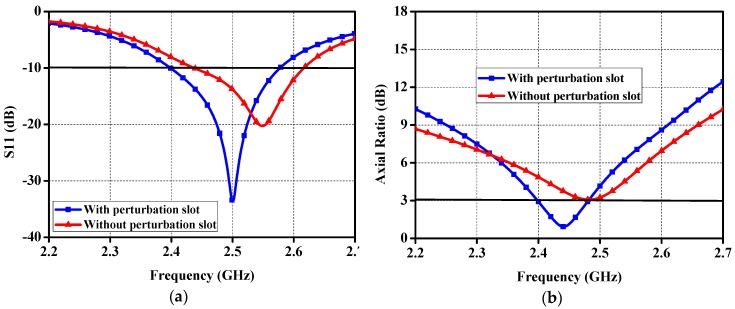
Performance of the proposed antenna with/without perturbation slots: (**a**) Reflection coefficient; and (**b**) AR.

**Figure 9:**
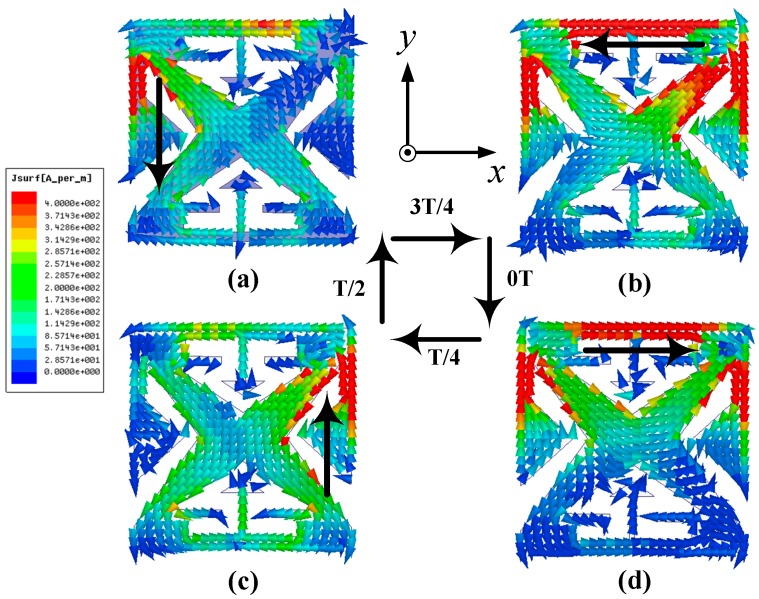
Current distributions on the patch at: (**a**) *t* = 0T; (**b**) *t* = T/4; (**c**) *t* = T/2; and (**d**) *t* = 3T/4.

As shown in Figure 2, perturbation slots are introduced in the patch to strengthen the CP performance. With reference to Figure 8, it can be seen that the small slots take critical role in the impedance matching and circular polarization. For the purpose of visualizing how the circular polarization is generated, the simulated surface current distributions on the patch at 2.45 GHz for four moments of 0T, T/4, T/2, and 3/4T are demonstrated in Figure 9. With the increment of time by a step of T/4, the currents rotate clockwise, transmitting LHCP waves in the boresight direction.

### 3.3. Parameter Studies

**Figure 10:**
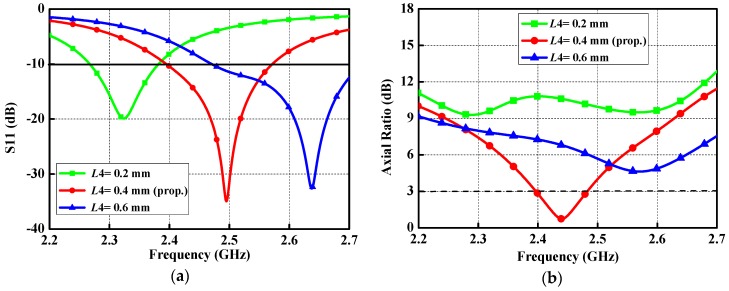
Performance of the proposed antenna with different *L*4 values: (**a**) Reflection coefficient; and (**b**) AR.

**Figure 11:**
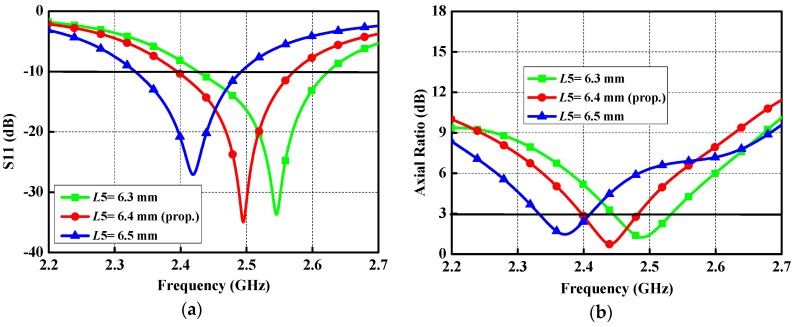
Performance of the proposed antenna with different *L*5 values: (**a**) Reflection coefficient; and (**b**) AR.

To obtain available guidelines for the practical design of the proposed antenna, various important parameters that can influence the return loss and axial ratio at the boresight direction are examined. As a key parameter is studied, the other parameters are kept constant. The width *L*4 of the high impedance microstrip line shows the crucial influence on the reflection coefficient and AR, due to its effect on the current distributions on the patch of the proposed antenna. As exhibited in Figure 10, the return loss and axial ratio are sensitive to different *L*4 values. A reasonable axial ratio at 2.45 GHz can be achieved when *L*4 is set to be 0.4 mm. If *L*4 becomes larger or smaller, there is a drastic influence on the AR. Figure 11 demonstrates the effect of tuning *L*5 on the performance of the proposed antenna. With reference to the curves in the figure, a linear increase in *L*5 (from 6.3 mm to 6.5 mm) will result in shifting the resonance down to the lower frequency and deviating the CP towards the lower band.

### 3.4. Safety Consideration

When the proposed antenna is implanted into the human body, specific absorption rate (SAR) for safety concerns should be evaluated. The IEEE C95.1-2005 standard limits the SAR average over any 10 g of tissue in the shape of a cube to less than 2 W/Kg (SAR 10g, max ≤ 2 W/kg) [26]. Therefore, through calculation, the maximum 10-g averaged SAR value is 81.5 W/kg at 2.45 GHz on condition that the power delivered to the proposed antenna is set to be 1 W, meaning that the delivered power should be below 24.5 mW to meet the IEEE C95.1-1999 standard.

## 4. Experimental Results

**Figure 12:**
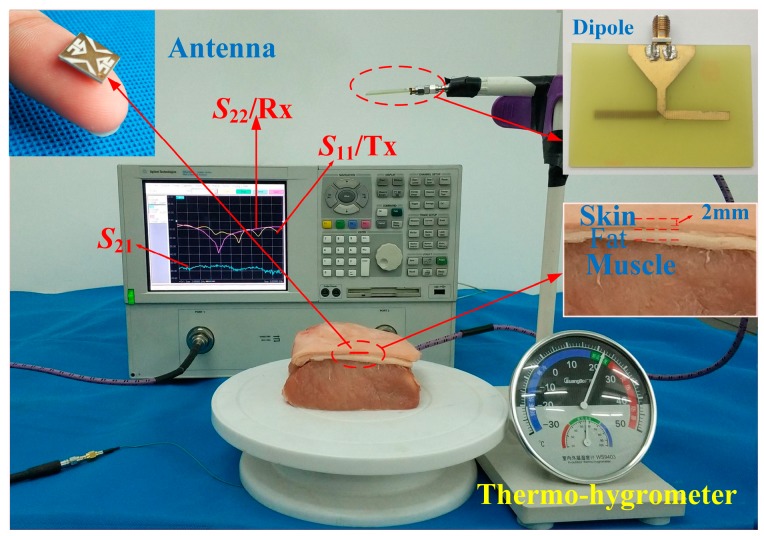
Photograph of the fabricated antenna prototype and measurement setup.

**Figure 13:**
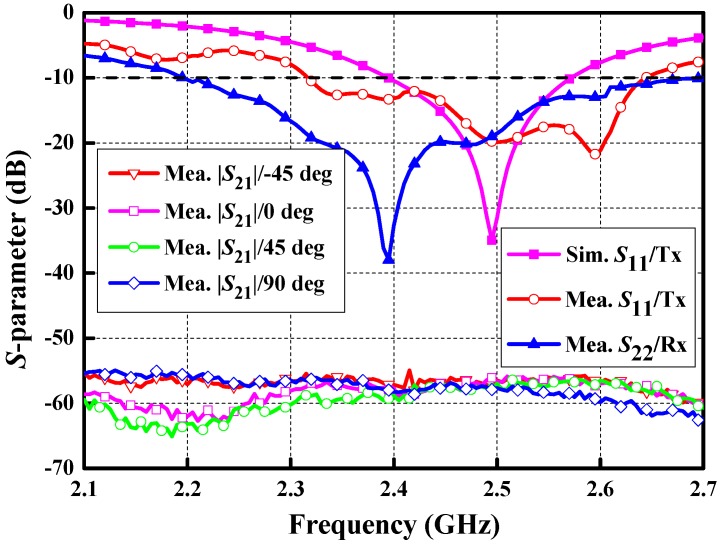
Measured S-parameters of the proposed antenna.

In order to validate the design strategy, the prototype of the proposed implanted antenna was fabricated and assembled. The measurement environment surrounding the proposed antenna is a piece of fresh streaky pork (shortly after slaughter) comprised layers of skin, fat, and muscle. Figure 12 shows the photos of fabricated antenna and measurement setup. The S-parameters of the antenna against frequency are measured with the aid of an Agilent N5230A vector network analyzer (VNA) (Keysight Technologies, Santa Rosa, CA, USA). The simulated and measured S-parameters are shown in Figure 13. The measured bandwidth for S11 < −10 dB is 12.9%, covering 2.32 to 2.64 GHz. There is a slight difference between the simulated and measured results mainly due to the tolerances in the fabrication process and measurement. A linearly polarized dipole, working as a receiver, is placed 150 mm away from the proposed antenna to testify the CP property of the proposed antenna. The S21 between the proposed antenna and the dipole was measured when the dipole was placed at the phi = −45°, 0°, 45°, and 90°, respectively. With reference to Figure 13, there is a maximum deviation only up to 3 dB for the measured S21, proving the CP property of the proposed antenna.

## 5. Conclusions

This article has numerically designed and experimentally studied a novel miniaturized single-fed circularly-polarized microstrip patch antenna at the ISM band of 2.40–2.48 GHz. By introducing reactive loading to form slow wave effect on the radiator and etching V-shaped slots on the main patch to lengthen the current flow path, a miniaturized antenna with the dimensions of 9.2 mm × 9.2 mm × 1.27 mm can be obtained. The radiations of the proposed antenna show a left-handed circular polarization when we adjust the sizes of geometrical structure. The prototype of the proposed implantable antenna has been implemented. The agreements between the simulated results and ex vivo measured ones have been reached.

As illustrated in Table 3, the performance of the proposed antenna is not perfect. Whereas, a trade-off has been reached. Compared with [5], although the dimensions of the proposed antenna are bigger, its AR bandwidth is broadened. The proposed antenna has less efficiency than the work in [21], but it has smaller dimensions and bigger AR bandwidth; compared with the art [23], the proposed antenna has compact dimensions with higher AR bandwidth; compared with [27], the proposed antenna has much compact dimensions with high efficiency. Hence, based on the slow wave effect, this novel implantable CP antenna is designed and miniaturized with the advantages of great size reduction and high polarization purity. The merit performance of the proposed implantable antenna shows the great potential in the application of biomedical telemetry, such as subcutaneous real-time glucose monitoring. 

## Figures and Tables

**Table 1:** Dielectric properties of different tissues at 2.45 GHz.

Tissues	Thickness (mm)	*ε* *_r_*	*σ* (S/m)
Skin	4	38.0	1.46
Fat	4	5.28	0.1
Muscle	50	52.7	1.74

**Table 2:** Dimensions of the proposed antenna parameters (unit: mm).

Symbol	Value	Symbol	Value	Symbol	Value
*L*	9.0	*W*	0.5	*L*1	4.6
*L*2	1.8	*L*3	0.6	*L*4	0.4
*L*5	6.4	*L*6	0.8	*L*7	2.6
*L*8	2.2	*L*9	0.9	*d*	3.5
*g*	0.1	*H*1	1.8	*H*2	1

**Table 3:** Comparison of the proposed antenna with prior art.

Ref.	Dimensions (mm × mm × mm)	Bandwidth		Peak Gain (dBi)
		(S11 < −10 dB)	(AR < 3 dB)	
[5]	8.5 × 8.5 × 1.27 (92 mm^3^)	2.32–2.62 GHz (~12.2%)	2.42–2.48 GHz (~2.4%)	−17
[21]	10 × 10 × 1.27 (127 mm^3^)	2.36–2.55 GHz (~7.7%)	2.44–2.48 GHz (~1.6%)	−22
[23]	π × (5.5)^2^ × 1.27 (~120 mm^3^)	2.31–2.51 GHz (~8.3%)	2.42–2.48 GHz (~2.49%)	−22.7
[27]	10 × 10 × 1.27 (127 mm^3^)	2.35–2.50 GHz (~6.2%)	2.36–2.56 GHz (~8.13%)	−27.2
This work	9.2 × 9.2 × 1.27 (107 mm^3^)	2.39–2.57 GHz (~7.2%)	2.39–2.48 GHz (~3.7%)	−24.8

## References

[1] Kiourti A., Nikita K.S. (2012). A review of implantable patch antennas for biomedical telemetry: Challenges and solutions. IEEE Trans. Antennas Propag. Mag..

[2] Soontornpipit P., Furse C.M., Chung Y.C. (2004). Design of implantable microstrip antenna for communication with medical implants. IEEE Trans. Microw. Theory Tech..

[3] Damis H.A., Khalid N., Mirzavand R., Chung H., Mousavi P. (2018). Investigation of epidermal loop antennas for biotelemetry IoT applications. IEEE Access..

[4] Neihart N.M., Harrison R.R. (2005). Micropower circuits for bidirectional wireless telemetry in neural recording applications. IEEE Trans. Biomed. Eng..

[5] Liu X.Y., Wu Z.T., Fan Y., Tentzeris E.M. (2017). A miniaturized CSRR loaded wide-beamwidth circularly polarized implantable antenna for subcutaneous real-time glucose monitoring. IEEE Antennas Wirel. Propag. Lett..

[6] Shah S.A.A., Yoo H. (2018). Scalp-implantable antenna systems for intracranial pressure monitoring. IEEE Trans. Antennas Propag..

[7] Hall P.S., Hao Y. (2012). Antennas and Propagation for Body-Centric Wireless Communications.

[8] Huang F.J., Lee C.M., Chang C.L., Chen L.K., Yo T.C., Luo C.H. (2011). Rectenna application of miniaturized implantable antenna design for triple-band biotelemetry communication. IEEE Trans. Antennas Propag..

[9] Duan Z., Guo Y.X., Xue R.F., Je M., Kwong D.L. (2012). Differentially-fed dual-band implantable antenna for biomedical applications. IEEE Trans. Antennas Propag..

[10] Kiourti A., Nikita K.S. (2012). Miniature scalp-implantable antennas for telemetry in the MICS and ISM bands: Design, safety considerations and link budget analysis. IEEE Trans. Antennas Propag..

[11] Merli F., Bolomey L., Zurcher J.F., Corradini G., Meurville E., Skrivervik A.K. (2011). Design, realization and measurements of a miniature antenna for implantable wireless communication systems. IEEE Trans. Antennas Propag..

[12] Warty R., Tofighi M.R., Kawoos U., Rosen A. (2008). Characterization of implantable antennas for intracranial pressure monitoring: Reflection by and transmission through a scalp phantom. IEEE Trans. Microw. Theory Tech..

[13] Xu L.J., Guo Y.X., Wu W. (2014). Miniaturized dual-band antenna for implantable wireless communications. IEEE Antennas Wirel. Propag. Lett..

[14] Izdebski P.M., Rajagopalan H., Rahmat-Samii Y. (2009). Conformal ingestible capsule antenna: A novel chandelier meandered design. IEEE Trans. Antennas Propag..

[15] Liu Y., Chen Y., Lin H., Juwono F.H. (2016). A novel differentially fed compact dual-band implantable antenna for biotelemetry applications. IEEE Antennas Wirel. Propag..

[16] Xu L.J., Guo Y.X., Wu W. (2012). Dual-band implantable antenna with open-end slots on ground. IEEE Antennas Wirel. Propag. Lett..

[17] Liu C., Guo Y.X., Xiao S. (2012). Compact dual-band antenna for implantable devices. IEEE Antennas Wirel. Propag. Lett..

[18] Liu W.C., Chen S.H., Wu C.M. (2009). Bandwidth enhancement and size reduction of an implantable PIFA antenna for biotelemetry devices. Microw. Opt. Technol. Lett..

[19] Jung Y.H., Qiu Y., Lee S.B., Shih T.Y., Xu Y., Xu R., Lee J., Schendel A.A., Lin W., Williams J.C. (2016). A compact parylene-coated WLAN flexible antenna for implantable electronics. IEEE Antennas Wirel. Propag. Lett..

[20] Duan Z., Guo Y.X., Je M., Kwong D.L. (2014). Design and in vitro test of a differentially fed dual-band implantable antenna operating at MICS and ISM bands. IEEE Trans. Antennas Propag..

[21] Liu C.R., Guo Y.X., Xiao S.Q. (2014). Capacitively loaded circularly polarized implantable patch antenna for ISM band biomedical applications. IEEE Trans. Antennas Propag..

[22] Liu C., Guo Y.X., Xiao S.Q. (2014). Circularly polarized helical antenna for ISM-band ingestible capsule endoscope systems. IEEE Trans. Antennas Propag..

[23] Li R., Guo Y.X., Zhang B., Du G.H. (2017). A miniaturized circularly polarized implantable annular-ring antenna. IEEE Antennas Wirel. Propag. Lett..

[24] Li J.M., Chang T.H., Liu X.Y. A compact circularly polarized antenna for in-body wireless communications. Proceedings of the 2017 IEEE International Symposium on Antennas and Propagation & USNC/URSI National Radio Science Meeting.

[25] Chi P., Waterhouse R., Itoh T. (2011). Antenna miniaturization using slow wave enhancement factor from loaded transmission line models. IEEE Trans. Antennas Propag..

[26] (2006). IEEE Standard for Safety Levels with Respect to Human Exposure to Radio Frequency Electromagnetic Fields, 3 kHz to 300 GHz, IEEE Standard C95.1-2005.

[27] Yang Z.J., Xiao S.Q., Zhu L., Wang B.Z., Tu H.L. (2017). A Circularly polarized implantable antenna for 2.4-GHz ISM band biomedical applications. IEEE Antennas Wirel. Propag. Lett..

